# Comparison of fecal pooling methods and DNA extraction kits for the detection of *Mycobacterium avium* subspecies *paratuberculosis*

**DOI:** 10.1002/mbo3.318

**Published:** 2015-12-15

**Authors:** Akiko Mita, Yasuyuki Mori, Tetsuo Nakagawa, Tomoko Tasaki, Katsuo Utiyama, Hitomi Mori

**Affiliations:** ^1^ Hygienics DivisionNational Livestock Breeding CenterNishigoNishi‐ShirakawaFukushima961‐8511Japan; ^2^ Division of Bacteriology and ParasitologyNational Institute of Animal HealthTsukubaIbaraki305‐0856Japan; ^3^ National Livestock Breeding CenterOkazaki StationOkazakiAichi444‐3161Japan

**Keywords:** Commercial DNA extraction kits, IS900, *Mycobacterium avium* subspecies *paratuberculosis*, pooled fecal samples, real‐time PCR, screening test

## Abstract

The aim of the study was to develop a sensitive method using quantitative real‐time polymerase chain reaction (qPCR) with pooled fecal samples for the screening of Johne's disease (JD). Manufacturer‐specified and our new pooling method in combination with five commercial kits for DNA extraction and purification were compared. Different volumes of pooled fecal suspensions were tested, and the results were compared for individual samples and three pool sizes (5, 10, and 50 samples); each of the fecal suspensions, which were prepared from healthy dairy and beef cattle was spiked with 0, 10, 100, or 1000 cultured *Mycobacterium avium* subspecies *paratuberculosis* (MAP) organisms or was mixed with fecal suspensions from experimentally infected cattle. The MAP DNA detection proportion with our pooling method in combination with Johne‐Spin kit (Fasmac, Japan) was 100% for all models and all pool sizes, except for the low shedder model with a pool size of 50. There was no loss of sensitivity in pools of 10 subjects or less by using the new method. These results suggest that new method is a sensitive, practical, and cost‐effective screening test for the detection of MAP‐infected cattle and the monitoring of JD‐free herds.

## Introduction

Paratuberculosis, also known as Johne's disease (JD), is caused by *Mycobacterium avium* subspecies *paratuberculosis* (MAP) and is an important alimentary infection of ruminants. The disease has a worldwide distribution, and it economically hinders dairy (Losinger 2005; Stott et al. 2005) and beef (Bhattarai et al. 2013) production. JD control is based on two fundamental strategies – testing and culling (TC) and vaccination (Bastida and Juste 2011).

The TC strategy depends on the diagnosis of MAP in infected and shedding cattle and removing them from the herd as soon as possible; however, the drawbacks of current diagnostic methods make this strategy difficult. Fecal culture is the “gold standard” for the diagnosis of JD, but it is costly and takes as long as 16 weeks (Collins 1996). A cost‐ and labor‐saving measure is to pool fecal samples of individual animals, but results still require several months, and the sensitivity achieved with pooled samples is much lower than that with individual samples, depending on the level of shedding (Whittington et al. 2000; Schaik et al. 2003; Dhand et al. 2010; Messam et al. 2010). Real‐time quantitative polymerase chain reaction (qPCR) of feces from individual subjects has gained popularity for rapid detection of shedding animals, with sensitivity and specificity comparable to those of fecal culture (Bögli‐Stuber et al. 2005; Douarre et al. 2010). However, this method is more costly and complicated than fecal culture. Enzyme‐linked immunosorbent assay (ELISA) is commonly used as a rapid and low‐cost screening serological test, but it has low sensitivity during the early stage of infection (Sweeney et al. 1995; Clark et al. 2008; Alinovi et al. 2009; Aly et al. 2014).

A new JD screening test that is time‐, labor‐, and cost‐saving and that has high sensitivity and specificity especially during early stage of infection is required for JD control strategies. A newly introduced pooled fecal qPCR test has been considered to satisfy these requirements (Aly et al. 2012). However, reports on pooled fecal sample qPCR tests are scarcer than those on individual fecal qPCR tests (Taddei et al. 2004; Leite et al. 2012). In addition, because feces has been attributed to the difficulty of removing PCR inhibitors (Monteiro et al. 1997; Thornton and Passen 2004), in a general pooling fecal method, feces or fecal suspensions are diluted to avoid increasing concentration of PCR inhibitors in pooled sample. However, it has the potential problems of decreased test sensitivity due to the dilution effect of sample pooling. Here, to develop a new pooled fecal qPCR test, manufacturer‐specified fecal pooling protocol and our new pooling protocols in combination with various commercial kits for DNA extraction and purification were compared.

## Material and Methods

### Samples and kits tested

In Experiment 1, a total of 1320 individual fecal samples were collected from 650 dairy and 670 beef cattle at the National Livestock Breeding Center (NLBC), Japan. At NLBC, all samples were confirmed negative for MAP DNA by using a combination of Johne‐Spin Kit (Fasmac, Kanagawa, Japan) and the MAP insertion sequence (IS) 900 qPCR in pooled 10 subjects. These methods were described in “Pooling and DNA extraction protocols for each kit” and “PCR analysis,” respectively. Negative fecal suspensions were prepared individually, pooled, and then divided into various volumes to simulate an individual sample and pooled samples of 5, 10, and 50 cattle. All samples were then sent to the National Institute of Animal Health (NIAH), Japan, where each negative sample was spiked with 0, 10, 100, or 1000 cultured MAP organisms in triplicate to simulate individual and pooled fecal samples containing feces from negative, low, moderate, and high MAP shedders. The spiked samples were coded for anonymity at NIAH and sent back to NLBC for use in Experiment 1. DNA extraction and qPCR were performed at NLBC; the data were sent to NIAH, and verified at NIAH. A commercial protocol for fecal pooling and DNA extraction by kit A (Tetracore MAP Extraction System, Tetracore Inc., MD) was compared with our in‐house protocol for fecal pooling followed by DNA extraction using kit B (Johne‐Spin kit), kit C (MagMax Total Nucleic Acid Isolation kit, Applied Biosystems, CA), and kit D (QIAamp stool DNA Mini kit, Qiagen GmbH, NW, Germany).

In Experiment 2, at NLBC, fecal suspensions prepared from cattle that were experimentally infected with MAP were diluted 5‐, 50‐, and 500‐fold with negative fecal suspension (e.g., one part of positive fecal suspension was mixed with 4, 49, or 499 part of negative fecal suspension) to simulate samples from moderate, low, and very low MAP shedders and were then mixed with various volumes of the pooled negative suspension for a comparison of kit B and kit E (ZR fecal DNA MiniPrep, Zymo Research Corp., CA). The mixed samples and the original negative suspension (negative control) were sent to NIAH. These samples were coded for anonymity at NIAH and sent back to NLBC, where the DNA extraction performances of kit B and kit E were compared as described earlier.

### Preparing known‐positive suspensions

Known‐positive suspensions were prepared from pooled negative fecal suspensions that were spiked with various amounts of MAP (Experiment 1) and fecal samples from experimentally infected cattle (Experiment 2).

In Experiment 1, the MAP reference strain ATCC 19698 was cultured on Herrold's egg yolk medium containing mycobactin J. The MAP organisms were harvested and suspended in saline, counted under the microscope after filtering the suspension through a 5‐*μ*m membrane filter to remove clumping of MAP, and spiked into the pooled negative suspension at NIAH, as described earlier.

In Experiment 2, two fecal samples from experimentally infected cattle (#63 and #65) were provided from NIAH, both of them were high MAP shedders with 10–100 colony forming units (CFU) per medium tube and their fecal MAP DNA amount detected by the IS900 qPCR were 39.2 pg and 19.1 pg per 0.1 g of feces, respectively. These MAP DNA quantities were determined by the qPCR analysis described in “PCR analysis” of Material and Methods section. At NLBC, positive fecal suspensions were prepared by diluting individually 5‐, 50‐, and 500‐fold with the pooled negative suspension, as described earlier, to simulate moderate (>10 CFU per media tube), low (1–10 CFU per media tube), and very low (<1 CFU per media tube) MAP shedders.

### Pooling and DNA extraction protocols for each kit

**Figure 1:**
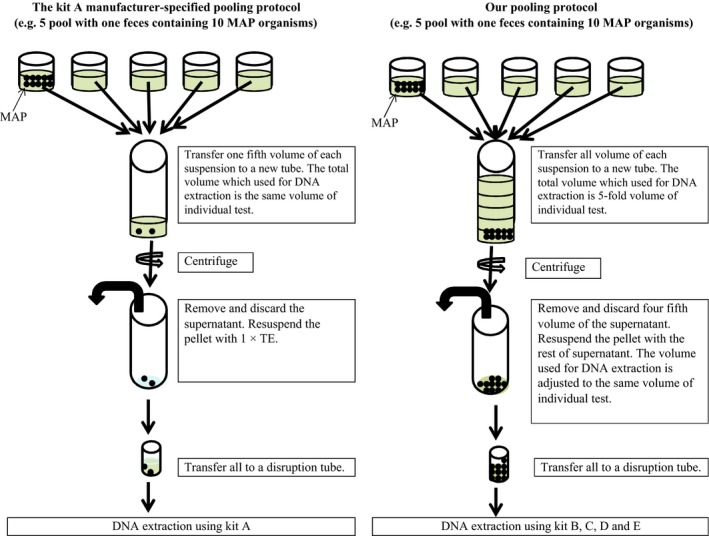
The kit A manufacturer‐specified and our fecal pooling protocol. The kit A protocol as general pooling protocol is that a fixed sample volume is used for DNA extraction, therefore which means those the *Mycobacterium avium* subspecies *paratuberculosis* (MAP) concentration in the fecal samples decreased and PCR inhibitors concentration in the fecal samples is as same as individual samples by the pooling process. Our fecal pooling protocol is the higher sample volume used for the DNA extraction and the resultant increased pooled sample volume, therefore which means those PCR inhibitors in the fecal samples increased and the MAP concentration in the fecal samples is as same as individual samples by the pooling process.

The kit A manufacturer‐specified pooling protocol was used before DNA extraction with kit A, whereas our protocol for fecal pooling was used for all other kits (Fig. 1). For kit A, 35 mL of sterilized water was added to 2 g of fecal sample, and the sample was vortexed 15 sec, rocked 30 min, and incubated 30 min at room temperature without agitation. A defined volume of the resultant upper portion (20 mL divided by the pool size) was mixed with pooled negative suspension to give a final volume of 20 mL: for example, in the case of the simulation of a pooled sample from five cattle, 20 mL divided by 5 – 4 mL of the positive suspension was mixed with 16 mL of the pooled negative suspension. This mixture was centrifuged (2500*g* for 10 min). The supernatant was discarded. The pellet was resuspended in 1 mL of 1× TE (10 m mol L^−1^ Tris‐HCl, pH 8.0; 1 m mol L^−1^ EDTA) and transferred to a disruption tube supplied with kit A, and DNA was extracted according to the kit A protocol.

For kit B, 20 mL of sterilized water was added to 1 g of fecal sample, and the sample was vortexed for 30 min using a vial mixer (TAITEC VIX‐100, TAITEC Co., Ltd, Saitama, Japan) and incubated for 30 min at room temperature without agitation. For individual samples, 1 mL of each suspension was transferred directly to the disruption tube provided with kit B, and DNA was extracted according to the kit B protocol. For pooled samples, the resultant suspension was mixed with pooled negative suspension to give a final volume in a new tube, and after centrifugation (900*g* for 30 min), all but 1 mL of the supernatant was discarded. For example, in the case of samples with a pool size of five, the total volume that was centrifuged was 5 mL (1 mL of positive suspension plus 4 mL of pooled negative suspension), and 4 mL of the supernatant was discarded. Each pellet was resuspended in the 1 mL of remaining supernatant and transferred to the disruption tube provided with kit B, and DNA was extracted according to the kit B protocol.

For kit C, 1 mL of sterilized phosphate‐buffered saline was added to 0.3 g of fecal sample, which was then vortexed for 3 min and centrifuged (100*g* for 1 min). For individual samples, 175 *μ*L of each supernatant was transferred directly to the disruption tube provided with kit C, and DNA was extracted according to the kit C protocol. For pooled samples, the resultant supernatant was mixed with pooled negative supernatant to give a final volume in a new tube and centrifuged (900*g* for 30 min), all but 175 *μ*L of supernatant was discarded. For example, in the case of samples with a pool size of 5, the total volume that was centrifuged was 875 *μ*L (175 *μ*L of positive supernatant plus 700 *μ*L of pooled negative supernatant), and 700 *μ*l of the supernatant was discarded. Each pellet was resuspended in the 175 *μ*L of remaining supernatant and transferred to the disruption tube provided with kit C, and DNA was extracted according to the kit C protocol.

For kit D, 2 mL of kit‐provided buffer was added to 0.2 g of fecal sample, the sample was vortexed for 1 min, and then 1.6 mL of each suspension was transferred to a new tube and heated at 95°C for 5 min, vortexed for 15 sec, and centrifuged (20,000*g* for 1 min). For individual samples, 1.2 mL of each supernatant was transferred to new tube directly, and DNA was extracted according to the kit D protocol. For pooled samples, the resultant supernatant was mixed with pooled negative supernatant to give a final volume in a new tube and centrifuged (900*g* for 30 min), all but 1.2 mL of supernatant was discarded. For example, in the case of samples with a pool size of 5, the total volume that was centrifuged was 6 mL (1.2 mL of positive supernatant plus 4.8 mL of pooled negative supernatant), and 4.8 mL of the supernatant was discarded. The pellet was resuspended in the 1.2 mL of remaining supernatant and transferred to a new tube, and DNA was extracted according to the kit D protocol.

For kit E, 750 *μ*L of kit‐provided lysis solution was added to 0.15 g of fecal sample in a disruption tube provided with the kit E, and the sample was vortexed for 5 min and centrifuged (7000*g* for 1 min). For individual samples, 400 *μ*L of each supernatant was transferred directly to a spin filter in a collection tube provided with the kit E, and DNA was extracted according to the kit E protocol. For pooled samples, the resultant supernatant was mixed with pooled negative supernatant to give a final volume in a new tube and centrifuged (900*g* for 30 min), all but 400 *μ*L of supernatant was discarded. For example, in the case of samples with a pool size of 5, the total volume that was centrifuged was 2 mL (400 *μ*L of positive supernatant plus 1.6 mL of pooled negative supernatant), and 1.6 mL of the supernatant was discarded. The pellet was resuspended in the 400 *μ*L of remaining supernatant and transferred to a spin filter in a collection tube provided with the kit E, and DNA was extracted according to the kit E protocol.

### PCR analysis

To detect MAP‐specific target genes, qPCR analysis of the DNA extracted with all kits was performed with primers specific to the MAP IS900, as described previously (Kawaji et al. 2007). In brief, a uracil DNA glycosylase reaction was conducted at 37°C for 10 min and DNA polymerase was activated at 95°C for 15 min, and 45 cycles of PCR amplification with denaturation at 95°C for 30 sec and annealing–extension at 68°C for 1 min. The total reaction volume was 25 *μ*L, with 2.5 *μ*L of DNA sample, 12.5 *μ*L of master mix (QuantiTect SYBR Green PCR kit, Qiagen GmbH, NW, Germany), 9.75 *μ*L of distilled water (DNase‐ and RNase‐free), 0.25 units of uracil‐DNA glycosylase (Uracil‐DNA Glycosylase, New England BioLabs Inc., MA), and 500 n mol L^−1^ of each primer. A sample was considered positive if at least one of the duplicate samples had the appropriate melting temperature (*T*_m_) and threshold cycle (*C*_t_) values in the dissociation curve analysis. MAP DNA concentrations were calculated according to the procedure described previously (Kawaji et al. 2007).

For DNA samples extracted with kit A, a second qPCR analysis for DNA extracted with kit A was performed with the use of PCR kit F (Vet Alert Johne's Real‐Time PCR, Tetracore Inc., MD) according to the kit protocol, because the manufacturer of kit A recommends the use of kit F provided by the same company. Kit F detects MAP‐specific hspX gene. Each sample was run in single. A sample was considered positive when it had an appropriated *C*_t_ value, even if the result appeared to cross the threshold after the cutoff value that is defined in the protocol.

In addition, a second qPCR analysis for DNA extracted with kit C was performed with the use of PCR kit G (VetMAX MAP Real‐Time PCR Screening kit, Applied Biosystems, CA), because the manufacturer of kit C recommends use of kit G (provided by the same company). A sample was considered positive when it had a *C*_t_ value for MAP DNA, even if the result appeared inconclusive or there was no signal for the internal positive control.

### Statistical analysis

The effect of the type of kit on the DNA yield or MAP detection proportion (i.e., the proportion of samples that were detected positive for MAP) was estimated by fitting the following linear model with the least square method by using Statsmodels:

$$Y_{\mathrm{ijkl}}=\mu +K_{i}+P_{j}+M_{k}+S_{l}+e_{\mathrm{ijkl}}$$

where *Y* is MAP detection proportion, *μ* is the overall mean, *K*_i_ is the effect of the kit (kit A [with PCR for IS900], kit A [with PCR kit F], kit B, kit C [with PCR for IS900]), kit C [with PCR kit G], or kit D), *P*_j_ is the effect of the pool size (1, 5, 10, or 50 head), *M*_k_ is the effect of the MAP shedder model (10, 100, or 1000 cultured MAP), *S*_l_ is the effect to the sample (1 to 72), and *e*_ijkl_ is the residual in Experiment 1; or where *Y* is the MAP DNA yield, *μ* is the overall mean, *K*_i_ is the effect to the kit (kit B or kit E), *P*_j_ is the effect of the pool size (1, 5, 10, or 50), *M*_k_ is the effect of the MAP shedder model (moderate, low, or very low), *S*_l_ is the effect to the sample (1 to 24), and *e*_ijkl_ is the residual in Experiment 2. All the coefficient and the 95% confidence interval were multiplied 100 for expressing as a percentage in Experiment 1. The effect of kit on MAP DNA yield at each MAP concentration was analyzed by one‐way analysis of variance and Tukey's multiple comparison tests in Experiment 1 by using Microsoft Excel.

## Results

In Experiment 1, fecal samples were spiked with known amounts of cultured MAP and the proportion of samples that were detected positive for MAP was compared among commercial kit A (with kit A protocol for fecal pooling) and three other commercial kits (kits B–D) (with the in‐house protocol for fecal pooling) (Table 1). There was marked variability in the MAP detection proportions achieved with the various kits, with the proportions ranging from 22% with kit D to 97% with kit B. Among the kits, kit A (with in‐house PCR using IS900) and kit B gave the highest proportions. The 95% confidence intervals of kit A (IS900) and kit B did not overlap with those of kit A (with PCR kit F), kit C (IS900), kit C (with PCR kit G), or kit D MAP DNA was not detected in any of the negative samples.

**Table 1:** Comparison of the *Mycobacterium avium* subspecies *paratuberculosis* (MAP) detection proportions for four commercial kits (A to D) by using fecal samples spiked with known amounts of MAP

MAP organisms per tube	Simulated pool size (no. of heads)	Kit A protocol for fecal pooling		Our protocol for fecal pooling			
		Kit A		Kit BIS900 PCR	Kit C		Kit DIS900 PCR
		IS900 PCR	Kit A specific PCR		IS900 PCR	Kit C specific PCR	
10	1	100%	67%	100%	0%	33%	0%
10	5	67%	0%	100%	0%	100%	0%
10	10	67%	0%	100%	0%	0%	0%
10	50	0%	0%	67%	0%	0%	0%
100	1	100%	100%	100%	100%	67%	33%
100	5	100%	67%	100%	67%	67%	0%
100	10	100%	67%	100%	33%	33%	0%
100	50	67%	0%	100%	0%	0%	0%
1000	1	100%	100%	100%	100%	100%	100%
1000	5	100%	100%	100%	100%	100%	100%
1000	10	100%	100%	100%	100%	100%	33%
1000	50	100%	0%	100%	100%	67%	0%
Total	Positive	83% (30/36)	50% (18/36)	97% (34/35^a^)	50% (18/36)	56% (20/36)	22% (8/36)
Coefficient		27.4	−3.3	40.3	−3.3	1.8	−29.0
95% Confidence interval		19.8 to 35.0	−10.9 to 4.3	32.5 to 48.0	−10.9 to 4.3	−5.8 to 9.4	−36.6 to −21.4

a One sample was omitted due to a defective spin column.

Numbers in parentheses are the number of matched samples per total positive samples.

**Figure 2:**
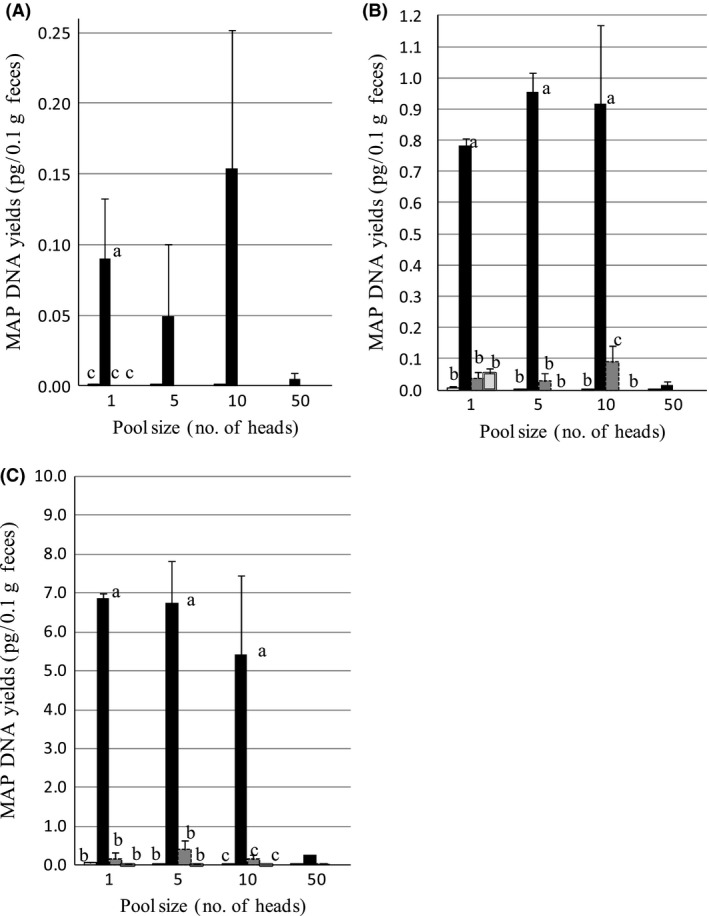
Comparison of the *Mycobacterium avium* subspecies *paratuberculosis* (MAP) DNA proportions of four commercial DNA extraction kits (kits A–D) for fecal samples spiked with cultured MAP. The final concentration of MAP organisms per tube was 10(A), 100(B), and 1000(C). DNA extraction kit was kit A (□), kit B (■), kit C (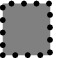), kit D (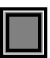). For all kits, IS900 PCR analysis was used. Values in groups marked “a” were significantly different from those in groups marked “b” (*P* < 0.01), and from those in groups marked “c” (*P* < 0.05), but values in groups marked “b” were not significantly different from those in groups marked “c” (*P* > 0.05). The number of samples per group was three expect the group of 1000 MAP organisms, kit B, and pool sizes of 50. The number of those groups was two because one sample was omitted due to a defective spin column.

The MAP DNA yields for kit A (with kit A protocol for fecal pooling) and kits B, C, and D (with our protocol for fecal pooling) were compared for fecal samples spiked with cultured MAP (Fig. 2). The DNA yield analysis was performed with in‐house PCR using IS900 on the same samples analyzed in Table 1 and an average of three samples in each case. For kit B, the average MAP DNA yields for pool sizes of 5 and 10 were similar to those obtained with individual samples; however, the average yield for a pool size of 50 was dramatically lower. Although the yields for kits A, C, and D were significantly lower than those for kit B.

In Experiment 2, to further examine differences in the efficiency of extraction and purification of MAP DNA in individual and pooled feces samples, the MAP DNA yields of kit B (the kit with the highest yield and MAP detection proportion in Experiment 1) and kit E (not examined in Experiment 1, see Discussion) were compared for fecal samples from experimentally infected cattle (Table 2). The samples were diluted 5‐, 50‐, and 500‐fold to simulate moderate, low, and very low MAP shedders. Both kits were used in combination with our protocol for fecal pooling, and MAP DNA was detected by IS900 qPCR analysis. Among the kits, there was marked variability in the proportion of samples that tested positive for MAP. The proportion was 100% with kit B and 71% with kit E, and the 95% confidence interval of kit B did not overlap with that of kit E. For individual samples, both kits provided MAP DNA yields above the limit of detection for all models (i.e., the very low, low, and moderate shedder models, respectively); and the average MAP DNA yields of the two kits were similar. For pool sizes of 5–50, MAP DNA was detected in all samples extracted with kit B, but not only some samples from the low and very low shedder models when kit E was used; in addition, for pooled samples, the average MAP DNA yields of kit E were in the order of one‐tenth lower than those of kit B.

**Table 2:** Comparison of the *Mycobacterium avium* subspecies *paratuberculosis* (MAP) DNA yield of two DNA extraction kits (kits B and E) with feces from experimentally infected cattle.^a^

Simulated pool size (no. of heads)	Sample		MAP status	Average MAP DNA yield (pg per 0.1 g feces)	
	ID	Dilution factor		Kit B	Kit E
1	#63	5×	Moderate	17.114	13.363
1	#65	5×	Moderate	2.863	3.064
1	#63	50×	Low	1.177	0.944
1	#65	50×	Low	0.344	0.145
1	#63	500×	Very low	0.077	0.044
1	#65	500×	Very low	0.113	0.085
5	#63	5×	Moderate	14.855	1.119
5	#65	5×	Moderate	4.876	0.459
5	#63	50×	Low	1.724	0.021
5	#65	50×	Low	0.380	0.035
5	#63	500×	Very low	0.087	–
5	#65	500×	Very low	0.023	–
10	#63	5×	Moderate	2.626	0.078
10	#65	5×	Moderate	4.673	0.055
10	#63	50×	Low	0.725	0.046
10	#65	50×	Low	0.295	0.024
10	#63	500×	Very low	0.012	–
10	#65	500×	Very low	0.037	–
50	#63	5×	Moderate	0.456	0.029
50	#65	5×	Moderate	0.231	0.012
50	#63	50×	Low	0.054	0.026
50	#65	50×	Low	0.038	–
50	#63	500×	Very low	0.001	–
50	#65	500×	Very low	0.007	–
Total	Positive			100% (24/24)	71% (17/24)
Coefficient				1.047	−0.338
95% Confidence interval				0.395 to 1.699	−0.99 to 0.314

a For both kits, our protocol for fecal pooling and IS900 PCR analysis were used. Average MAP DNA yield: – = No product. MAP status: very low = <1 CFU per tube; low = 1–10 CFU per tube; moderate = >10 CFU per tube. Numbers in parentheses are the number of matched samples per total positive samples.

## Discussion

A variety of components in feces are known to inhibit PCR. The amount of these PCR inhibitors varies depending on individual animals and the day of sampling, because it may be linked to feedstuff (Monteiro et al. 1997). Here, to equalize the influence of PCR inhibitors, fecal suspensions were prepared individually from healthy cattle and then pooled and divided into different pool sizes. This pooled fecal preparation was used to compare all kits.

In Experiment 1, the combination of our protocol for fecal pooling and the commercial kit B protocol for DNA extraction gave the highest detection proportion of MAP DNA from all pooled fecal samples. The potential advantage of this protocol is the higher sample volume used for the DNA extraction and the resultant increased pooled sample volume. In this protocol, false‐negative results should be taken into consideration because of the increase in PCR inhibitors from fecal samples; however, the high detection proportion achieved with kit B suggests that this kit could efficiently remove PCR inhibitors from pooled fecal samples. In contrast, the possible disadvantage of the commercial kit A protocol is that a fixed sample volume was used for DNA extraction, which means that the MAP concentration in the fecal samples is diluted by the pooling process. Despite the increased levels of PCR inhibitors, the MAP DNA detection proportion with our pooling protocol in combination with kit B was 100% for all models and all pool sizes, except for the low shedder model with a pool size of 50 (detection proportion, 67%). The manufacturer of kit A recommends that it not be used for pool sizes greater than 5; however, the MAP kit A (kit F) lacked sensitivity for the low shedder model even for individual samples (detection proportion, 67%) and pool size of 5 (detection proportion, 0%).

The highest detection proportion and MAP DNA yields were obtained from kit B, followed by kit A, kit C, and kit D in this order. For kits B–D which were used in combination with our pooling protocol, the order of the detection proportions reflected the order of the average MAP DNA yields, when IS900 primers were used in the qPCR analysis. The results obtained here for kits A, C, and D are consistent with a previous report that compared the MAP detection proportion for individual fecal tests across commercial kits (Leite et al. 2012).

The fecal suspension protocol for kit E is unique. Because the feces are added directly to each disruption tube without dilution with water or buffer, the samples could not be spiked with cultured MAP organisms. Therefore, the performance of kit E could not compared with that of other kits in Experiment 1. For that reason, the detection proportion and average MAP DNA yields of kit B and kit E were compared by using feces of experimentally infected cattle in Experiment 2. The performance of kit B was the highest of kits A–D in Experiment 1, and the performance of kit E was the highest in the previous report, which examined individual samples (Leite et al. 2012). In a preliminary study, the average MAP DNA yield of kit E was slightly higher than that of kit B for individual samples. The yield of kit E was 100‐ to 1000‐fold that of kits A, C, and D (data not shown). Here, the performances of kit B and kit E were similar for individual samples. In contrast, for pooled samples, kit B detected MAP DNA in all models examined (moderate, low, and very low shedding), whereas kit E did not consistently detect DNA in the very low shedding models. In addition, for each model, the average MAP DNA yields of kit E for pooled samples were less than one tenth those of kit B. Taken together, these results suggest that PCR inhibitors from pooled feces strongly influenced the performance of kit E.

Our protocol of fecal pooling combined with DNA extraction using kit B showed the best performance in the qPCR test for detection of MAP DNA in pooled fecal samples. Furthermore, the results of this study indicate that DNA samples prepared using this protocol may be useful in the qPCR test without loss of sensitivity up to a pool of nine noninfected and one MAP shedding cattle. If a commercially available qPCR kit for the detection of MAP DNA was used for a pool size of 10, the total cost may be less than that of ELISA. In addition, it has been reported that the specificity and sensitivity of the qPCR test are extremely high (Bögli‐Stuber et al. 2005; Clark et al. 2008; Alinovi et al. 2009; Douarre et al.^2010^; Aly et al. 2012, 2014). These findings suggest that a pooled fecal qPCR test using a combination of our fecal pooling protocol, kit B DNA extraction, and IS900 PCR is a sensitive, practical, and cost‐effective screening test for the detection of MAP‐infected cattle and the monitoring of JD‐free herds.

## Conflict of Interest

None declared.
